# Delayed Perceptual Awareness in Rapid Perceptual Decisions

**DOI:** 10.1371/journal.pone.0017079

**Published:** 2011-02-17

**Authors:** Regina Gregori-Grgič, Monica Balderi, Claudio de'Sperati

**Affiliations:** Visuo-Motor Functions Lab, Univeristà Vita-Salute San Raffaele, Milano, Italy; Ecole Polytechnique Federale de Lausanne, Switzerland

## Abstract

The flourishing of studies on the neural correlates of decision-making calls for an appraisal of the relation between perceptual decisions and conscious perception. By exploiting the long integration time of noisy motion stimuli, and by forcing human observers to make difficult speeded decisions – sometimes a blind guess – about stimulus direction, we traced the temporal buildup of motion discrimination capability and perceptual awareness, as assessed trial by trial through direct rating. We found that both increased gradually with motion coherence and viewing time, but discrimination was systematically leading awareness, reaching a plateau much earlier. Sensitivity and criterion changes contributed jointly to the slow buildup of perceptual awareness. It made no difference whether motion discrimination was accomplished by saccades or verbal responses. These findings suggest that perceptual awareness emerges on the top of a developing or even mature perceptual decision. We argue that the middle temporal (MT) cortical region does not confer us the full phenomenic depth of motion perception, although it may represent a precursor stage in building our subjective sense of visual motion.

## Introduction

Simple perceptual decisions have become an important test-bed for theories on decision-making. Tasks such as identifying a target among distractors, or discriminating the direction of a noisy motion stimulus, or making a saccade to a target, have stimulated the development of a variety of theoretical models, which incorporated several behavioural and neurophysiological observations [1], [2], [3], [4]. An important outcome of these studies is the notion that neurons in parietal and frontal regions accumulate evidence over time until a given decision criterion is reached (rise-to-threshold mechanisms), fuelling the idea that these neurons implement probability “reasoning” to emit a verdict about the state of the world.

Perception, however, involves crucially also a phenomenic dimension, that is, a conscious subjective sensation, or perceptual awareness. In the visual domain, this is what we ordinary call “seeing”. Indeed, one of the most challenging goals for modern neurosciences is to understand how consciousness emerges out of non-conscious processes [5]. A way to address this issue is to measure the perceptual delay, which implies identifying when a sensory stimulation becomes a conscious percept [6], [7]. Some studies estimated the perceptual delay to be in the order of few tenths of seconds [7], [8], [9], but the kind of stimulus and task can affect significantly the processing time [10], [11]; Degraded or ambiguous stimuli, for example, may require much longer to be identified [12]. However, estimating the perceptual delay is not as easy as measuring response times, as a visuo-motor response may not tag the moment in which we become aware of a stimulus. Alternative approaches, such as taking the earlier difference of certain EEG events (e.g., the latency of evoked potentials, or the peak of induced gamma synchrony, [10], [13], [14]) when comparing conditions in which a given stimulus is either seen or unseen, may simply reveal specific precursors of conscious perception. Further, especially with degraded stimuli, conscious perception may not be an all-or-none phenomenon, but a continuum of clarity unfolding in time [6], [10], [15], so that the notion of a precise point in time at which the conscious sensation pops out may be too simplistic. Therefore, when dealing with the temporal dynamics of conscious perception, it is important to understand how perceptual awareness builds up over time.

**Figure 1:**
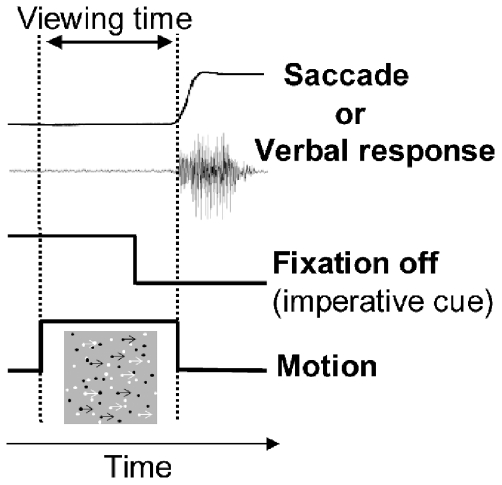
Stimulus and motion discrimination task. See Methods for details.

Despite the diffusion of studies on perceptual decision-making, and the number of hypotheses on how the decision signal accumulates over time [1], [2], [3], [4], [16], the temporal relation between the objective decision performance and the associated subjective sensation has never been investigated directly, perhaps also owing to the above difficulties. Here we addressed this issue by jointly assessing how visually-guided decisions and visual awareness develop in time after stimulus onset. We took advantage of degraded motion stimuli that entail a long processing time [12], [17], and sampled the accumulation of sensory evidence by forcing participants to make speeded discrimination of motion direction, with a saccade or a verbal response, at various points in time after stimulus onset, guessing if necessary (Figure 1). In each trial, observers rated the subjective visibility of global motion direction through a perceptual awareness (PA) scale.

## Materials and Methods

### Ethics statement

The study was conducted in accordance with the recommendations of the Declaration of Helsinki and the local Ethical Committee (“Comitato etico”, San Raffaele). Before the experiments, all participants signed the informed consent.

### Participants

Eight healthy observers volunteered in the main experiment (6 females and 2 males, with normal or corrected-to-normal vision, aged between 22 and 34, naive as to the purpose of the experiment except two authors). They received a book in return for the collaboration.

### Stimuli and task

Observers were seated in a darkened room about 60 cm in front of a computer screen (Sony Trinitron, 21 inches; frame-rate: 85 Hz; resolution: 800×600 pixels; background luminance: 19 cd·m^−2^), with the head resting on a forehead support. The stimuli (random dot kinematograms, Figure 1) were generated by a custom program written in MATLAB (6.5.0 R13, The MathWorks Inc., 2002, equipped with PsychToolbox 3), and consisted of 100 dots (diameter  = 0.2 deg), half black (luminance  = 0.2 cd·m^−2^) and half white (luminance  = 118 cd·m^−2^), moving linearly at a velocity of 5 deg·s^−1^, with a lifetime of ∼82 ms (7 frames), presented within a central square aperture (side  = 11 deg). At each lifetime cycle, the dots were regenerated in a different position within the screen aperture; part of them was assigned a randomly-chosen motion direction (random motion), while the remaining part kept moving in the same direction (coherent motion, either rightward or leftward). The proportion of the latter dots determined the coherence of the stimulus, which could be 0%, 15%, 30%, or 60%.

Each trial started with the presentation of a central fixation dot (diameter: 0.3 deg; luminance: 118 cd·m^−2^), followed by the motion stimulus after a random interval comprised between 1 and 2 s (Figure 1). The offset of the fixation dot was the imperative cue to emit immediately the response (direction discrimination task), either verbally (“right”/”left”) or with the eyes (in this case the instruction was to make a saccade in the direction of motion, to the right or left border of the stimulus pattern). The response terminated the stimulus within 4 video-frames. We manipulated the duration of the fixation dot, which was switched off 0.2, 0.5, 1 or 4 s after the onset of the motion stimulus. Throughout the text, this manipulation – an experimental factor – will be called “urgency”, to denote the fact that, especially at 0.2 and 0.5 s, the imperative cue forced observers to emit a sudden, early response. Higher urgencies corresponded to shorter durations of the fixation dot. Observers could respond before the offset of the fixation dot if they had perceived clearly the global motion direction, which could happen especially at the lowest urgency (4 s).

The imperative cue was quite effective in determining a prompt response with a remarkably small variability of response times, except at the lowest urgency (Table 1). Observes were informed that in many cases the global motion direction might not be seen at all, in which case the discrimination response must be a blind guess. Before the beginning of the experiments, participants got acquainted with the task, which required about 100–200 trials.

**Table 1:** Response times of the discrimination responses.

Response Times (ms)						
	Coherence = 15%		Coherence = 30%		Coherence = 60%	
Urgency	*Mean*	*S.E.M.*	*Mean*	*S.E.M.*	*Mean*	*S.E.M.*
200 ms	403	25	352	35	307	40
500 ms	314	25	194	60	109	70
1 s	226	58	−18	124	−230	142
4 s	−652	344	−1708	588	−2536	536

Data are relative to the imperative cue (offset of the fixation dot). Negative values indicate anticipation. S.E.M.: standard error of the mean across subjects.

In the control experiment (three additional participants, 2 females and 1 male, with normal or corrected-to-normal vision, aged between 22 and 27, naive as to the purpose of the experiment) the procedure was substantially the same as in the main experiment, except for the presence of a condition in which the perceptual judgment (actually a simpler yes/no task, see the Results section) was given upon the imperative cue. In another condition it was given after the discrimination response, i.e., at the end of the trial as in the main experiment. The two conditions were administered in different sessions (3 for each condition, 256 trials per session) in a counterbalanced order. Motion discrimination was performed only with verbal responses, and ocular fixation was monitored throughout the trial.

### Recording procedures

The real-time stimulus presentation was monitored by means of an analog photocell system, and the signal recorded together with the eye position traces and the verbal traces. Eye movements (horizontal component, monocular) were recorded through infrared oculometry (Dr. Bouis Oculometer, nominal accuracy <0.3 deg), while the verbal discrimination responses were recorded with a directional microphone and an amplifier. The analog signals were visualized in real time on an oscilloscope, sampled through and A/D converter (16 bit, 1000 Hz), and stored for subsequent analyses. In the verbal response experiments, trials containing saccades or accidental utterances were discarded, and re-presented at the end of the session. In the saccade experiments, trials containing saccades before the offset of the fixation dot, or small saccades (<1 deg), or ocular artifacts (e.g., eye blinks) were discarded, and re-presented at the end of the session.

### Rating task

In order to capture as reliably as possible the first-person, subjective quality of visual experience [18], we used a measure of perceptual awareness structured as a type I task with an absolute 5-points scale. Firstly, we wanted the perceptual judgment to target specifically the visibility of the motion stimulus (type I task: judging an external event [19]) rather than the confidence of the observer's own discrimination response (type II task: judging an internal event): Being confident of the response is not equivalent of being aware of the stimulus [20]. Secondly, we used a multi-point scale instead of a yes/no task to capture the graded nature of perceptual awareness [6], [10], [15], especially because we worked with degraded stimuli. The number of points was chosen to make the scale simple and comfortable for the observers: It has been shown that four categories are enough for the PA scale to be informative, while a high number of categories may be confusing [15]. Thirdly, we took caution to anchor the scale to the minimum and maximum absolute values [21], namely, null visibility and full visibility, and ensured that participants understood that the scale should represent a linear quantity. We insisted that the observers assigned a score of zero only in case of complete invisibility of global motion direction. The instructions to the participants, which were given in both written and colloquial form, were the following: 0 =  You didn't see at all the motion direction; 1 =  Between 0 and 2: you had a raw feeling of the motion direction; 2 =  Half-way point of the scale: you probably saw the motion direction; 3 =  Between 2 and 4: you saw the motion direction, but not too well; 4 =  You saw clearly the motion direction.

### Experimental design and data analyses

For each trial, the dependent variables were the actual viewing time (which in turn was determined by the urgency condition and the response time), the response direction, and the PA score. As for the independent variables, we used a randomized mixed design with 5 factors: motion direction (within-subjects factor, 2 levels: right/left), response modality (between-subjects factor, 2 levels: saccade/verbal response), stimulus coherence (within-subjects factor, 4 levels: 0%, 15%, 30%, 60%), urgency (within-subjects factor, 4 levels: 0.2 s, 0.5 s, 1 s, 4 s), repetition (N = 8), and block (N = 5). Each block consisted of a single experimental session with 256 trials (average duration: ∼1 hour, including pauses), for a total of 1280 trials per subject. Participants were invited to take pauses whenever they wished or felt tired. The five blocks were normally administered in different days.

By means of an interactive computer program we determined the onset of saccades (the moment at which the instantaneous horizontal eye velocity exceeded 30 deg·s^−1^ for more than 15 ms), and verbal responses (the moment at which the envelope of the vocal emission exceeded the noise level). At this stage, 87 trials (∼0.8% of the total) were eliminated due to residual artifacts that went unnoticed during the data acquisition phase.

Within the framework of Signal Detection Theory (SDT, [22]), the observers' sensitivity in the rating task was assessed through both area and distance measures. We used the non-parametric index A', which estimates the area under the ROC (Receiver Operating Characteristics) curve (AUC), as well as the distance index d'. The area under the ROC curve is 0.5 when the performance is at chance, while it is 1.0 when the performance is errorless. The corresponding values for d' are 0.0 and ∞. The response bias was assessed by calculating the location criterion c =  −½[z(H)+z(F)], which is independent of d'. We treated the rating task as a multiple yes/no detection task: For each observer, and for each coherence and urgency condition, we calculated hits (on signal trials: coherent motion) and false alarms (on noise trials: coherence  = 0%) over four response combinations. Given the five PA scores used in our experiment, there were four possible pairs of hits and false alarms rates. That is, PA scores greater than 0 were first considered to be ‘yes’ responses, while the 0 score was considered to be a ‘no’ response; next, PA scores greater than 1 were considered to be ‘yes’ responses, while PA scores less than 2 were considered to be ‘no’ responses, and so on, until encompassing all pairs of hits and false alarms rates. In the control experiment, in which the rating task was replaced by a yes/no task, d' was computed simply as z(H)–z(F). In the motion discrimination task d' was computed also as z(H)–z(F), where hits are the correct responses to an arbitrary stimulus direction (in our case rightward), and false alarms are the correct responses to the other stimulus direction. To adjust for extreme values, the entries in the contingency tables have been increased by 0.25 [23].

The degree of correlation between the performance in the discrimination task and subjective visibility was assessed with the non-parametric Kendall τ, which tested the trial-by-trial relationship between the correctness of the discrimination response (a binary variable) and the PA score [24]. The discrimination rate was tested against chance with the one-tail binomial test (alpha level  = 0.01). We used repeated-measures ANOVAs to test the significance of the discrimination rate, AUC, and PA scores (all values were z-transformed, with the latter previously re-scaled in the 0.5–1 range) over coherence (excluding 0%) and urgency conditions. Separate repeated-measures ANOVAs were used to assess the significance of d'. Pair-wise comparisons at each urgency and coherence conditions were tested with the paired Student *t*.

## Results

**Figure 2:**
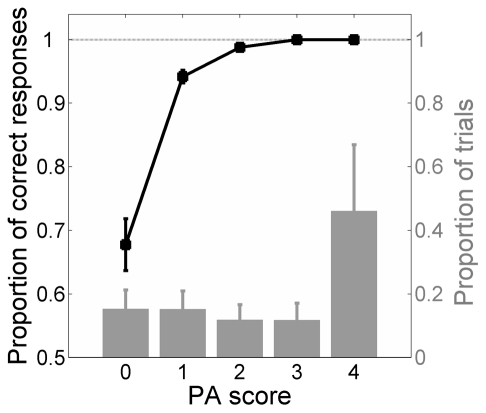
General relationship between motion discrimination and perceptual awareness. Black symbols: mean proportion of correct responses (chance level  = 0.5) at each PA score. The grey histogram represents the frequency distribution of PA scores. Data are collapsed over urgency and coherence (excluding coherence  = 0%). Bars: ± S.E.M. across subjects.

Overall, the discrimination rate was positively correlated with the PA score (τ = 0.342, p<0.001, Figure 2). Observers discriminated motion direction rather well (between 87.1% and 98.8% across subjects, excluding stimuli with coherence  = 0%), even at PA = 0, where the average discrimination rate was 68% (with no significant difference between saccadic and verbal response trials, 64% and 71%, respectively). The latter values were significantly higher than chance (p<0.001), suggesting that motion could be processed to a considerable degree even when its direction was subjectively invisible. The significantly better-than-chance performance at PA = 0 held true in 7 out of 8 participants, with discrimination rates comprised between 57% and 87%.

The correlation between motion discrimination and perceptual awareness seems to run counter the existence of a dissociation between decision and awareness [21]. However, while the significant correlation may indicate some common sensory processing, when discriminating motion observers may exploit the (same) sensory signal better than when making a perceptual judgment ([25]; see also [26]). Because processing degraded motion is time-consuming, it is possible that the discrimination decision and perceptual awareness increase jointly with both stimulus coherence viewing time, but with the former being systematically superior to the latter. As a consequence, it should take longer for perceptual awareness to build up.

**Figure 3:**
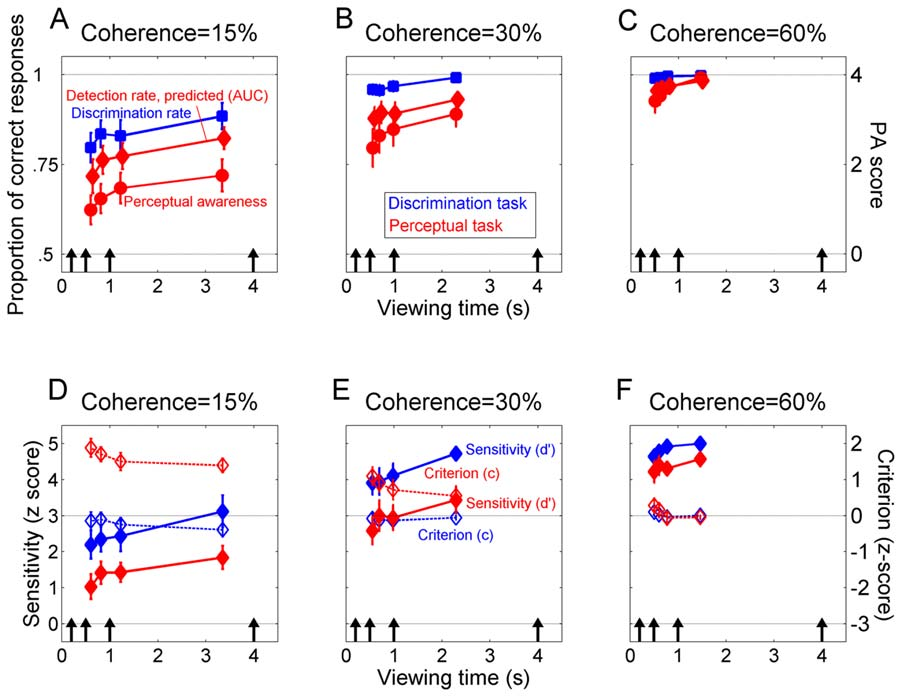
Time-course of discrimination capability and perceptual awareness. A–C: Effects of coherence and urgency on discrimination rate, perceptual awareness and AUC. Perfect discrimination (100%) corresponds to maximal AUC (A' = 1) and full visibility (PA = 4), while chance discrimination (50%) corresponds to zero-sensitivity (A' = 0.5) and null visibility (PA = 0). Note however that the PA scale is not linear with respect to the other two measures. The values are plotted on the horizontal axis in correspondence of the mean viewing time, and represent means ± S.E.M. across subjects. Black arrows: offset of the fixation dot in each urgency condition. Time zero represents stimulus onset. D–F: Effects of coherence and urgency on sensitivity and response criterion. Same convention as in A–C.

As urgency decreased – and viewing time increased – both the mean discrimination performance and the mean subjective visibility increased (Figure 3A–C; main effect of urgency on discrimination rate: p = 0.002; on PA score: p<0.001), but perceptual awareness tended to saturate well after the discrimination rate. About 600 ms after stimulus onset the percentage of correct responses at coherence  = 60% had already reached 99%, whereas perceptual awareness was clearly still increasing over time. The median values of PA were very similar to the mean values (not shown).

In order to compare the discrimination task and the rating task with the same metrics, we estimated the area under the ROC curve (AUC) in the rating task, which according to SDT corresponds to the proportion of correct responses that would be obtained should an unbiased observer had performed a 2-alternatives forced-choice (2afc) detection task (area theorem, [22]). The AUC increased with viewing time (main effect of urgency, p = 0.004), and remained always below the motion discrimination rate (main effect of task, p<0.001), until it saturated at the highest stimulus strength. Remarkably, a clear growth of AUC – as well as perceptual awareness – was still well visible in correct trials (83% of total trials, Supporting Figure S1), which means that selecting correct responses is not a sufficient criterion to ensure that awareness is already stabilized, even with relatively long viewing times.

The AUC is an intuitive measure because it expresses the detection performance in terms of percent correct. However, it assumes an unbiased observer. Moreover, our discrimination task was not a 2afc comparison task, but a 2afc identification task, as each trial contained only one stimulus [22], which may imply a lower performance. Therefore, depending on the amount of bias, the performance difference between the two tasks may not be fully reflected in the AUC-based analysis. We thus compared the two tasks also in terms of the distance metrics d' (Figure 3D–F), which estimates the system sensitivity to sensory signals independently of response criterion, and bias, which estimates the response criterion independently of sensitivity (see Methods). In the rating task, the sensitivity indicated the capability to separate rightward or leftward motion from noise, while in the discrimination task the sensitivity indicated the capability to separate rightward from leftward motion. Because both the discrimination and the rating tasks were performed on the same – single – stimulus, no √2 correction for 2-intervals vs. 1-interval task design was applied [27]. This analysis showed that d' increased with both viewing time and stimulus coherence (main effect of urgency, p<0.001; main effect of coherence, p<0.001), and in the rating task it remained lower than in the discrimination task (main effect of task, p<0.001, p<0.001, and p = 0.018 at coherence  = 15%, 30%, and 60%, respectively). Note that d' values in the order of 4–5 standard deviations indicate a very large separation between the internal distributions, and thus represent an extremely high performance; the more d' increases, the less the performance increases in terms of percent correct.

The response criterion is plotted in figure 3D–F. In the rating task positive values indicate the tendency to give low PA scores, and in the discrimination task positive values indicate a preference to respond “left”. In the rating task, the changes in the response criterion paralleled the changes in sensitivity, as it decreased with decreasing urgency and increasing coherence (p<0.002 in both cases). That is, top-down components tending to decrease the perceptual judgment were more prominent with shorter and weaker motion stimuli, when less external information is available. By contrast, the response criterion in the motion discrimination task was never significantly different from zero (always p>0.1, except at urgency  = 4 s and coherence  = 15%, p = 0.041, one-sample Student *t*).

We found no significant differences between the saccade and the verbal response trials (p>0.15 for every F-test containing the response modality as factor), suggesting that the mode of response is irrelevant in this kind of perceptual decision.

The pattern of result was very robust both within and between observers, despite the different inter-individual responsiveness to different stimulus coherences (Supporting Figure S2). Distinct time-courses of motion discrimination rate, AUC and perceptual awareness – as well as sensitivity and criterion – were also clearly visible when pooling the data for task performance instead of stimulus coherence (Supporting Figure S3).

**Figure 4:**
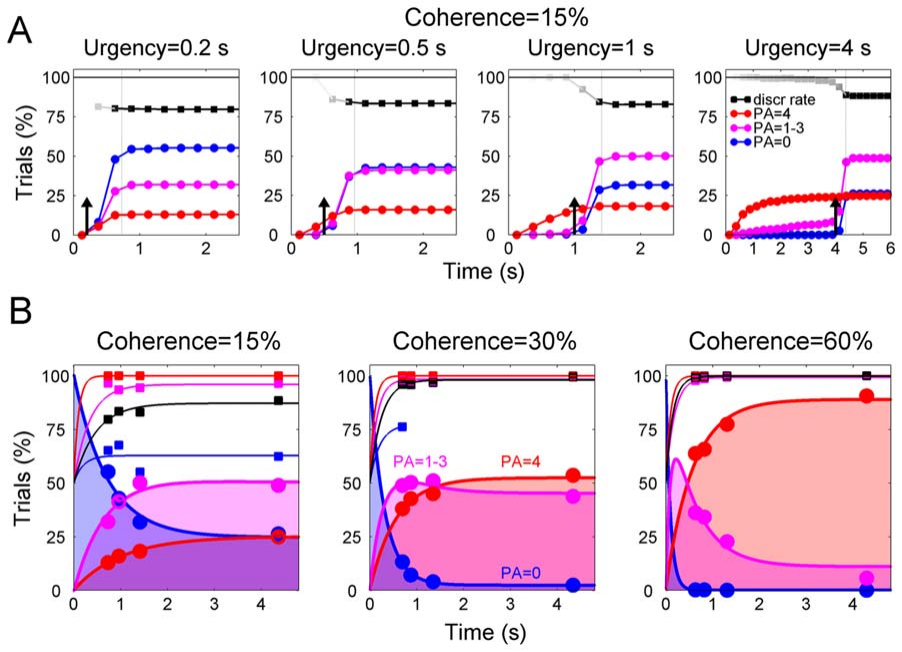
Cumulative frequency of PA scores over time (A) and visibility curves (B). A: Trials were subdivided in full visibility trials (PA = 4, red), intermediate visibility trials (PA = 1–3, magenta), and blind trials (PA = 0, blue). Time zero is motion onset. The curves with the squares represent the instantaneous motion discrimination rate, where the grey tone maps linearly the number of trials (total cumulative frequency distribution; white  =  no trials, black  = 100%). The vertical dotted lines indicate the moment at which the total cumulative trial frequency reached 95%, at which time the value of each cumulate frequency of PA scores was sampled to build the visibility curves illustrated in panel B. The short vertical arrows indicate the disappearance of the fixation dot (imperative cue) at each urgency condition. Data from all observers were pooled together. Bin width  = 250 ms. B: Visibility curves. Temporal evolution of the frequency of the three classes of PA scores (circles and thick curves). Also plotted is the time-course of discrimination rate for each class (squares and thin curves). The fitted curves for null and full visibility trials were obtained with a 3-parameters exponential function, while the curves for intermediate visibility were obtained by subtracting from 100 the sum of the instantaneous values of the functions of null and full visibility trials, so that the total instantaneous probability was always 1. We assumed that at the time of stimulus onset the blind trials would represent the totality of the potential responses (100%), with both the intermediate and full visibility trials being absent (0%). The fitted curves for the discrimination rate were obtained with a 2-parameters exponential function, under the assumption that at the time of stimulus onset the discrimination rate would be at chance (50%). The discrimination rate was computed only when the number of trials on which it was based was >10% of the trials in each urgency condition. The fitting models served only descriptive purposes. Time zero represents stimulus onset. Data from all observers are pooled together.

To describe in more details the temporal evolution of subjective motion visibility, we calculated also the instantaneous cumulative frequency of individual PA scores over time (Figure 4A). For simplicity, the trials with PA score between 1 and 3 were pooled into a single category of “intermediate visibility” (magenta). The other categories were “blind” trials (PA score  = 0, blue) and “full visibility” trials (PA score  = 4, red). We reconstructed the time-course of subjective visibility by plotting, for each urgency condition, the proportion of blind, intermediate visibility, and full visibility trials at the time the total cumulative frequency distribution reached 95%. The resulting visibility curves (thick lines, Figure 4B) depicted the rise of full visibility over time, and the fall of invisibility, with intermediate visibility first increasing and then decreasing. Note that, especially at 15% coherence, there was an initial time window after stimulus onset in which blind trials predominated. In these trials, the discrimination rate (blue squares) was better than chance (always p<0.001 except in one case), but the rapid fading of invisibility implied that fully unconscious motion processing was short-lived. Clearly, there must be a point in time where, by further decreasing response times, also the discrimination rate in blind trials would drop to chance level. Due to the lower bound of response times, we could not explore a closer temporal proximity of stimulus onset. However, the reconstructed curves give a reasonable idea of the very initial moments of the build-up of motion discrimination capability and perceptual awareness. Again, we found no substantial differences between saccades and verbal response trials (not shown).

Assessing the subjective motion visibility after the discrimination task might somewhat underestimate the visibility at the time of discrimination, because of possible masking effects of the visual transient at the cessation of the stimulus, or simply because of the delay between the two tasks. We checked this point in a control experiment, in which we asked observers to make the perceptual judgment at the time of the imperative cue. However, especially at higher urgencies, it was very difficult for the observers to rate on the 5-points scale the subjective visibility of the motion stimulus shortly after stimulus onset, as evidently this task requires a certain minimum time to be accomplished. Therefore, we replaced the perceptual task based on the 5-points rating with an easier y/n task, in which observers had just to respond verbally “yes” or “no” for “motion seen” or “motion unseen”, respectively. In a first condition observers had to give the y/n response at the time of the imperative cue (speeded y/n task). The performance in this task was compared to a condition in which the y/n response was given after motion discrimination (delayed y/n task), a condition that replicated the temporal structure of the main experiment, where the PA score was reported after the discrimination response. The two sensitivity curves (d') were almost overlapping in the two tasks (Supporting Figure S4), as well as the curves of the criterion (c), and no significant differences were detected in sensitivity (always p>0.26 for every F-test containing the task as factor), criterion (always p>0.27), or viewing time (always p>0.10). Pair-wise comparisons of sensitivity between the two tasks also failed to reach significance (always p>0.065). These data suggest that in the main experiment the reported motion visibility (PA scores) indeed reflected the subjective visibility at the time of the discrimination response.

## Discussion

This study has documented a higher performance in the motion discrimination task compared to the rating task. That is, observers identified the global motion direction with a forced-choice procedure better than expected on the basis of the reported subjective visibility of the same motion stimulus. Because of the long integration time of our motion stimuli, this performance difference translated into a longer buildup of perceptual awareness relative to the buildup of motion discrimination capabilities. The buildup of perceptual awareness was associated to a slow rise of full visibility trials after stimulus onset that gradually replaced the short-lasting blind trials and the more long-lasting intermediate visibility trials. Observers showed good or even perfect discrimination before perceptual awareness reached a steady-state, even when blind trials were still predominant, in which case a fleeting “blindsight-like” mode [24], [28] contributed a modest but significant discrimination performance (even though patients with lesions to V1 do not normally discriminate the direction of random-dot motion in their blind field [29]). These data suggest that speeded decisions about motion direction can exploit degraded visual information better than – and thus before – conscious perception.

Task differences may contribute to the superiority of motion discrimination. For example, depending on the similarity of the stimulus attributes actually used in the two tasks, the separation between rightward and leftward motion signals underlying the discrimination response may be larger than the separation between motion and noise signals underlying the PA rating (see [22], p. 191). However, it is doubtful that observers could take advantage of this difference of sensory information with only one stimulus available, as no stimulus comparison was made. We predict that the same results would be obtained if observers had to make speeded discrimination of motion and noise stimuli, instead of rightward and leftward stimuli.

The pattern of results was the same regardless of whether motion discrimination was accomplished with a saccade or a verbal response, which ruled out that speeded responses depended on a dedicated visuo-motor reflex by-passing the circuitry for visual perception. This could reflect the fact that our saccadic task was not primarily a motor-oriented task (the saccadic component was incidental), in which case automatic sensorimotor responses faster than visual perception could indeed be expected [8], [30]. Also, the relatively long timescale of the task excluded that motion discrimination was carried out in a single pass of feed-forward cortical processing, which would act within ∼100–200 ms [31], [32]. Rather, we were facing a more general dissociation between intuitive or even blind decisions and the rather sluggish perceptual awareness [4], a dissociation that tended to disappear once full subjective visibility was attained, at which time motion discrimination became perceptually-driven.

Speeding up the discrimination response by imposing urgency to the task, was paid with a decrease of both discrimination performance and perceptual awareness. While the former effect is a manifestation of the well-known speed-accuracy trade-off [33], the latter effect implies an analogous trade-off between speed and awareness, but shifted in time. This is interesting because it suggests that, regardless of which particular compromise between speed and accuracy is set in a difficult perceptual decision, subjective visibility is always sacrificed, even when discrimination accuracy is maximal, except for very long viewing times. Indeed, in some observers the subjective visibility rose slowly over time, whereas the discrimination performance reached 100% almost immediately after stimulus onset (Supporting Figure S2).

### Assessing perceptual awareness

This study addressed the issue of conscious perception. Consciousness is in the first place regarded, both in popular and scientific psychology, as a subjective, private phenomenon. As such, it appears to be ultimately inaccessible to a third-person approach. This notion of consciousness corresponds roughly to what the philosopher Ned Block termed phenomenal consciousness [34]. The distinction between phenomenal consciousness and access consciousness (i.e., a conscious state that is not entirely private) justifies the distinction between a purely phenomenological approach to consciousness, and the approach typical of scientific psychology and neurosciences. However, this passage is far from obvious [35], and enduring disputes about the nature of consciousness persist, as also witnessed by the polysemy of the term consciousness, and the variety of related terms and qualifiers. Throughout the paper we use the terms “awareness”, “consciousness”, “phenomenal”, “sensation”, “subjective visibility”, as synonyms, and are intended to denote the first-person visual experience of “seeing”, whereas the term “perception” is used in a rather liberal way. The difficulty to assess conscious perception in controlled experiments, in particular, gave rise to endless methodological discussions as to the best approach to consciousness, and what counts as index of conscious experience – e.g., direct vs. indirect, objective vs. subjective measures [18], [25], [36].

In order to capture as directly as possible the first-person, subjective quality of visual experience, while at the same time keeping an objective stance, we took as a primary index of visual consciousness the explicit visibility judgments given by the observers [18]. We opted for a simple PA scale that accommodates different degrees of subjective visibility without constraining visual perception in the Procrustean bed of the conscious/unconscious dichotomy (see Methods), an issue that is particularly cogent under uncertainty conditions. Although a perceptual judgment may be regarded to involve meta-cognitive processes distinct from the “genuine” phenomenal fact, we preferred here to avoid such a clear-cut separation, and considered simple perceptual judgments as the basic unit of measurable, subjective perceptual experience. That is, we tried as much as possible to go to the heart of conscious visual perception by tagging the natural notion of “seeing” – regardless of its fuzzy or illusory nature – from the point of view of the observer.

A posteriori, the observers' perceptual judgments turned out to be both reliable and veridical. They were reliable, because the pattern of results was very systematic, both within and between observers. They were veridical, because when the stimulus was pure noise (coherence  = 0%), the reported visibility of global motion direction was almost null (mean PA scores ranging from 0.08 to 0.16 over urgency levels, not shown), and because especially at the highest stimulus coherence and long viewing time perfect visibility was easily attained (PA score  = 4). That is, observers used the entire PA scale without difficulty.

Note that despite our observers were carefully instructed to assign a zero score only when they didn't see at all the global stimulus direction, it is possible in principle that a zero visibility score could in fact tag sometimes cases in which visibility was not truly “null”, but simply not worth a score of one. However, a visual experience that for a normal observer is not even worth a minimum visibility judgment should be labeled “blindness”, and therefore we took the trials with PA = 0 as trials in which observers were blind to motion direction in a substantial way (blind trials).

By using SDT we aimed at providing a common currency for quantifying the performance in the two tasks, and at assessing the contribution of top-down and bottom up components in the perceptual judgment (criterion and sensitivity), without however pretending that perceptual awareness is entirely captured by a single objective quantity [36]. Yet, we believe that combining a somewhat qualitative approach (e.g., Figure 4) with a quantitative analysis based on the transformation of the visibility ratings into an equivalent detection performance (e.g., Figure 3) is a simple way to capture satisfactorily the subjective quality of perceptual experience while at the same time remaining within a robust methodological framework (see also [37]).

### Perceptual decisions can precede awareness

When considering the temporal dynamics of perception, the superiority of motion discrimination capability over subjective motion perception has an interesting consequence: awareness and discrimination become dissociated in time, i.e., perceptual awareness lags discrimination, in the sense that it takes longer to reach the same performance level and to stabilize. This does not imply strictly serial processes, as the processes underlying discrimination and awareness can coexist in time (race model). Note that we are not suggesting to take the saturation of perceptual awareness as the temporal marker of the perceptual delay; Rather, its gradual buildup suggests that the notion of a precise point in time where conscious perception is realized may be too strict, at least with our degraded motion stimuli.

That perceptual awareness is more sluggish than motion discrimination may appear somewhat unsettling, as we tend often to assume that conscious perception precedes decision. However, phenomena such as blindsight [24], [28] and unconscious perception [18], [25] suggest that automatic decisions are indeed possible under certain conditions. In more ordinary contexts, many sensory-driven actions, as well as the stimuli that originated them, pass mostly unnoticed, as when driving or in sports. Similarly, we incessantly decide where to make the next gaze shift, despite poor or null awareness of peripheral - and sometimes also central - visual information [38], [39]. Awareness may just follow.

In general, a slow dynamics of perceptual awareness may reflect the need to go beyond the contingencies of a continuously changing world. For example, if the temporal dynamics of perceptual awareness were strictly tuned to the timing of exploratory eye movements, we would probably see the world as a rapid sequence of snapshots, one for each fixation period. Indeed, we recently found that a spontaneous increase of the saccadic latency from ∼200 to ∼500 ms resulted in a progressive increase of their susceptibility to an illusory mislocalization effect [8], which suggested that perceptual awareness of the target position lagged short-latency saccades (blind saccades). Also, very fast responses to natural objects (∼100 ms, [40]), and even more so the amazingly fast color discrimination capability recently discovered in the monkey (∼30 ms, [41]), call for an automatic process that may not wait for conscious perception [31]. Thus, transient dissociations, long or short, between decision and perception may be the rule, rather than the exception, during everyday life, in the sense that our sluggish perceptual awareness can lag visually-guided motor responses [8], [9], [30], action selection [42], perceptual decisions [41], and even intuitive choices [43]. Another reason why awareness should be delayed is that, in order to experience the world as unitary despite the various asynchronies deriving from multiple sensory (and also non-sensory) signals, a buffer system is needed that accommodates all possible asynchronies; The readout of a buffered system is by necessity delayed. A general picture thus arises that depicts awareness as a post-hoc construct emerging on the top of a developing or even mature decision, both when the stimulus is internal, as in unconscious initiation of a voluntary motor act [7], or external, as in the present study.

In disentangling decision from conscious perception, our study warns against an indiscriminate use of monkeys' saccadic eye movement as a proxy for conscious visual perception (e.g., for what monkeys “see”), even when accuracy is rewarded [1], [2], [3]. More generally, our findings indicate that, especially when time is an issue, objective forced-choice responses may not provide a full account of visual perception, as the perceptual decision can be taken when the formation of perceptual awareness is still underway. Note that in our data there was a non-negligible proportion of intermediate visibility trials in which observers anticipated the imperative cue, which is a hint that even well-trained observers may tend to respond somewhat automatically before perceptual awareness is stabilized. In the less corticalized monkey, automatic visuo-motor decisions could be even more pronounced. Thus, because selecting correct responses is a too lenient criterion to ensure that perceptual awareness is already stabilized, it would be important to measure directly the degree of monkey's perceptual awareness, an issue that however is theoretically far from obvious [35], [44]. Building upon past work [45], [46], [47], it might perhaps be possible to train monkeys to pair the motion discrimination response with a simplified rating task for perceptual awareness. In principle, comparing the buildup of perceptual awareness and perceptual decisions in monkeys would permit to assess how much their visual awareness is flattened to the swift dynamics of a perceptual decision.

### Seeing global motion: neural correlates of conscious vision

How can a perceptual decision be taken when perceptual awareness is still developing? At least for global motion direction, the answer may lay in the particular mechanism that is thought to regulate the formation of the decision signal from the underlying visual signal. Several findings, both in humans and monkeys, indicate area MT as a crucial node for motion processing (see [48] for review). In recent years a growing body of data have disclosed also the role of area LIP (Lateral Intraparietal) in perceptual decisions involving motion stimuli (see [3] for review). When a monkey is instructed to make an eye movement to report the direction of a random-dot kinematogram, neurons in LIP pick up sensory evidence, presumably from MT, and integrate it for some hundreds of ms until a decision bound is reached, and an oculomotor command issued. Importantly, the decision is reached even when the stimulus is still available or the response procrastinated, because LIP neurons exploit only the initial part of the discharge of MT neurons [49], [50]. Thus, monkey LIP seems to work as a device that implements a relatively quick rise-to-threshold mechanism for various types of visuo-motor responses when a perceptual decision is required [51]. In this way, the decision is ready even though MT neurons are still processing the motion input. Note that structures other than LIP could be involved in motion discrimination when the response is verbal, perhaps as part of a circuit for more abstract decision-making [4], although the fact that we found a pattern of results virtually identical for saccades and verbal responses suggests that similar mechanisms may be at play.

Perceptual awareness would instead require a longer processing of visual signals. At a first sight, the long discharge of MT neurons, that outlasts the perceptual decision, could be thought of as the neural basis for the formation of visual awareness for motion. However, in the anaesthetized monkey, MT neurons respond promptly to the onset of random dot motion, and the information rate saturates very quickly (within ∼100 ms, [52]. Obviously, such automatic, almost time-invariant response cannot give rise to perceptual awareness, and is rather involved in smooth pursuit initiation. Moreover, in the awake monkey the sensitivity to global motion of individual MT neurons, while increasing over time (for about 2 seconds), is comparable to the sensitivity of motion discrimination of both monkeys and humans, as assessed through coherence threshold [53]. Yet, we showed that, at least in humans, the sensitivity in the discrimination task is higher than the sensitivity in the rating task. Thus, if the same holds for monkeys, the sensitivity of MT neurons would be too high to be compatible with perceptual awareness. Therefore, a distinct, sub-optimal readout mechanism should be hypothesized, perhaps through the pooling a sub-population of MT neurons containing a large amount of correlated noise that collectively have a lower signal-to-noise ratio than those feeding LIP for a perceptual decision [54]. Thus, in this scenario neither MT nor LIP are sufficient for the formation of perceptual awareness, as at least an additional readout mechanism would be required, exhibiting integration properties compatible with the slow buildup of perceptual awareness. Clearly, an alternative scenario is that in the monkey motion discrimination and perceptual awareness are not dissociated, in which case monkeys may have only an immediate, faint subjective sense of motion, in principle entirely accountable by the activity in MT/LIP.

In humans, the contribution of area MT to motion awareness remains a matter of speculation [55], although findings such as that a bilateral damage to this region causes motion blindness [56], or that implied and imagined motion activate MT [57], [58], or that MT exhibits spatiotopic properties [59], suggest an important role in high-level motion processing. To shed light on the role of human MT in the buildup of awareness of global motion, it would be important to know whether its temporal integration properties are compatible with the long buildup of perceptual awareness or whether they just comply with the relatively quick dynamics of perceptual decisions. Because temporal summation in human MT does not appear to be very long-lasting when tested with stimuli similar to those employed in our study (M.C. Morrone, personal communication), it is doubtful that this cortical structure can fully support the slow buildup of perceptual awareness for motion. Thus, it would seem that MT can be considered a high-level area as far as global motion processing is concerned, but a low-level area as far as the associated subjective quality of motion vision is concerned.

The above considerations do not exclude that neural circuits in MT and/or LIP may take part in an early phase of perceptual elaboration as a precursor of conscious perception. MT could contribute to form a faint feeling of motion, as in motion imagery [57], [58], [60], perhaps reinforced by multiple connections with earlier striate and extrastriate areas (especially human V3A, whose response to motion is remarkable [61]), or by the recruitment of other cortical circuits [13], [30], [62]. Decision-related activity in LIP may contribute to form the subjective confidence in the discrimination performance [46], which depends on an internal evaluation of both one's own decisional capability and the degree of awareness of the stimulus [19]. The involvement of a sensori-motor area such as LIP in visual perception would be generally in line also with current views of embodied cognition and motor theories of perception [63].

In sum, among the mosaic of visual areas activated by global motion in humans [64], only those supporting temporal integration properties compatible with the slow buildup of perceptual awareness would candidate as a specific neural correlate of consciousness, sufficient to confer us the full phenomenic depth of motion perception. Clearly, a less simplistic view is that awareness is a large-scale, distributed property [13], in which case no single cortical structure may exhibit a macroscopic activation that co-varies on its own with the conscious percept.

### Conclusions

Our study has documented a remarkable capability of identifying the direction of degraded motion, with a saccade or a verbal response, at a time after stimulus onset when motion is still subjectively invisible or poorly visible. This sort of “transient blindsight” suggests that many ordinary perceptual decisions – not necessarily motor – can be effectively taken when our sluggish conscious representation of the world is still a void or a faint impression. As a consequence, forced perceptual decisions may not tell the entire story about visual perception, especially when time is an issue.

## Supporting Information

Figure S1 **Time-course of perceptual awareness for correct responses.** Conventions as in Figure 3 of the main text.
(TIF)

Figure S2 **Individual performance.** Same as Figure 3 of the main text, but the data are presented for each individual observer (‘S#’), and for each response modality (‘s’ for saccade trials, ‘v’ for verbal response trials). The thickness of the box around the plots tags the grouping criterion used to pool the data for task performance (see Figure S3). Each layer of the figure contains the data relative to one observer and one response modality.
(TIF)

Figure S3 **Time-course of discrimination capability and perceptual awareness, pooled by task performance.** For this, we first identified the stimulus coherence at which discrimination rate, AUC and perceptual awareness saturated at all urgency conditions (e.g., coherence  = 60% for S1s in Figure S2). The “High coherences” panel included data relative to the immediately lower coherence (panels labeled with a black thick box, Figure S2). The “Low coherences” panel included data relative to the next lower coherence (gray boxes of Figure S2). If no joint saturation was attained (in S4s, S3v, S4v), the data from coherence  = 60% were included in the “High coherence” group. Only seven subjects formed the “Low coherences” group, because in subject S2v the lowest tested coherence (15%) pertained to the “High coherences” group.
(TIF)

Figure S4 **Control experiment.** Sensitivity (d', continuous lines) and criterion (c, dotted lines) in the yes/no task. The red symbols (delayed y/n) refer to a condition in which the y/n response was given after the motion discrimination task, a condition that mimicked the main experiment. The yellow symbols (speeded y/n) refer to a condition in which the same observers were forced to give the y/n response immediately after the imperative cue (in place of the motion discrimination response). The mean discrimination rate in the delayed y/n condition was 77%, 89%, 96%, respectively for 15%, 30%, and 60% coherence. Same conventions as in Figure 3D–F of the main text.
(TIF)
