# Supplementary figures and images for: Delayed Perceptual Awareness in Rapid Perceptual Decisions
Source: PLoS One. 2011 Feb 17;6(2):e17079. doi: 10.1371/journal.pone.0017079 (PMC3040746; doi:10.1371/journal.pone.0017079)

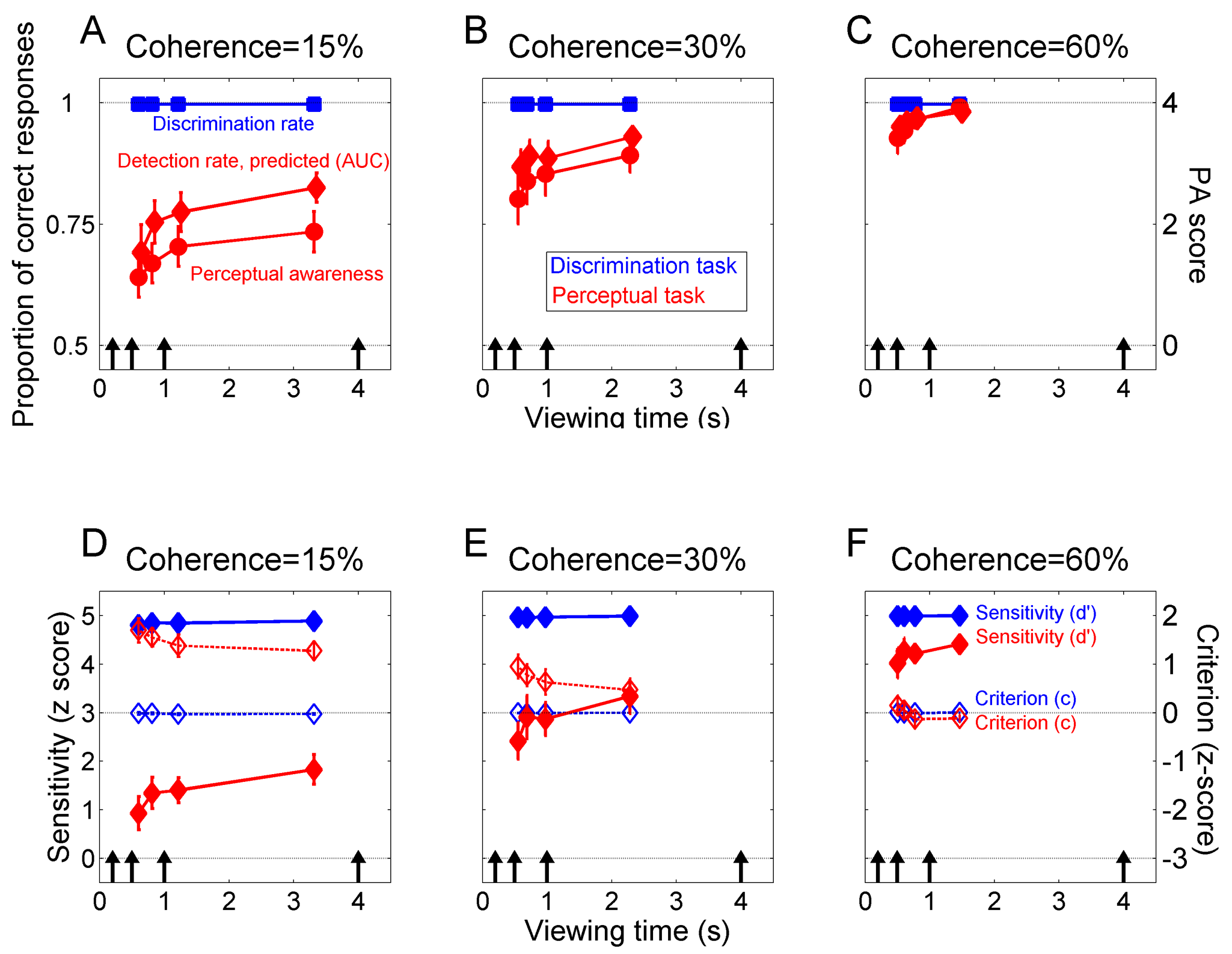

Supplement: Figure S1 — Time-course of perceptual awareness for correct responses. Conventions as in Figure 3 of the main text. (TIF) [file pone.0017079.s001.tif]

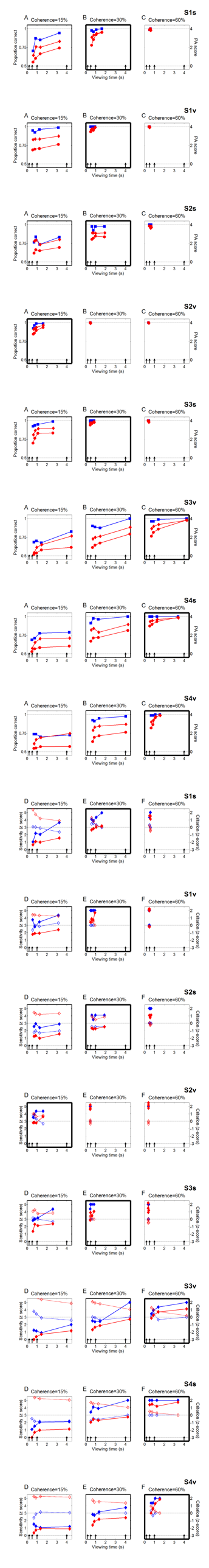

Supplement: Figure S2 — Individual performance. Same as Figure 3 of the main text, but the data are presented for each individual observer (‘S#’), and for each response modality (‘s’ for saccade trials, ‘v’ for verbal response trials). The thickness of the box around the plots tags the grouping criterion used to pool the data for task performance (see Figure S3). Each layer of the figure contains the data relative to one observer and one response modality. (TIF) [file pone.0017079.s002.tif]

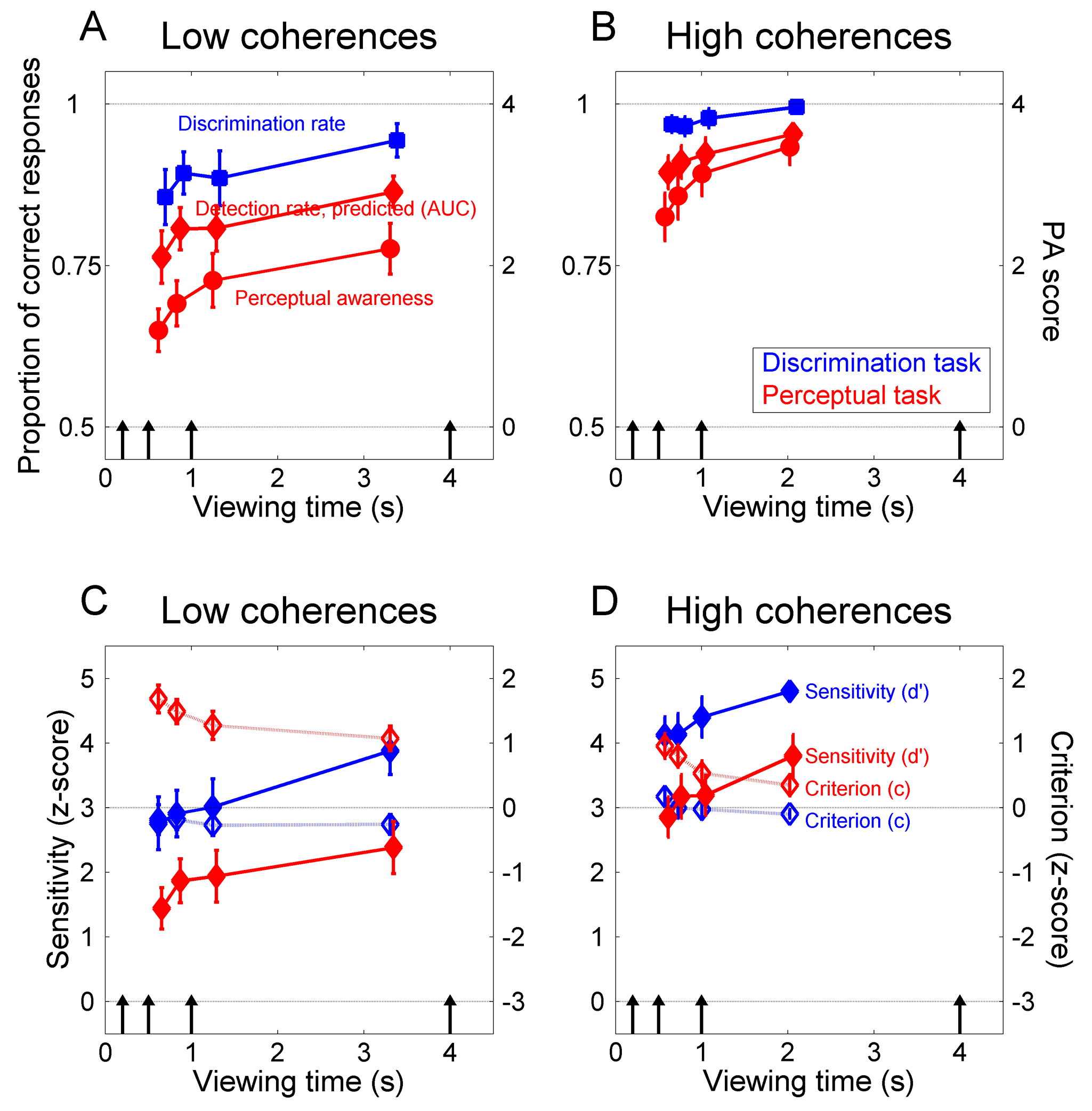

Supplement: Figure S3 — Time-course of discrimination capability and perceptual awareness, pooled by task performance. For this, we first identified the stimulus coherence at which discrimination rate, AUC and perceptual awareness saturated at all urgency conditions (e.g., coherence = 60% for S1s in Figure S2). The “High coherences” panel included data relative to the immediately lower coherence (panels labeled with a black thick box, Figure S2). The “Low coherences” panel included data relative to the next lower coherence (gray boxes of Figure S2). If no joint saturation was attained (in S4s, S3v, S4v), the data from coherence = 60% were included in the “High coherence” group. Only seven subjects formed the “Low coherences” group, because in subject S2v the lowest tested coherence (15%) pertained to the “High coherences” group. (TIF) [file pone.0017079.s003.tif]

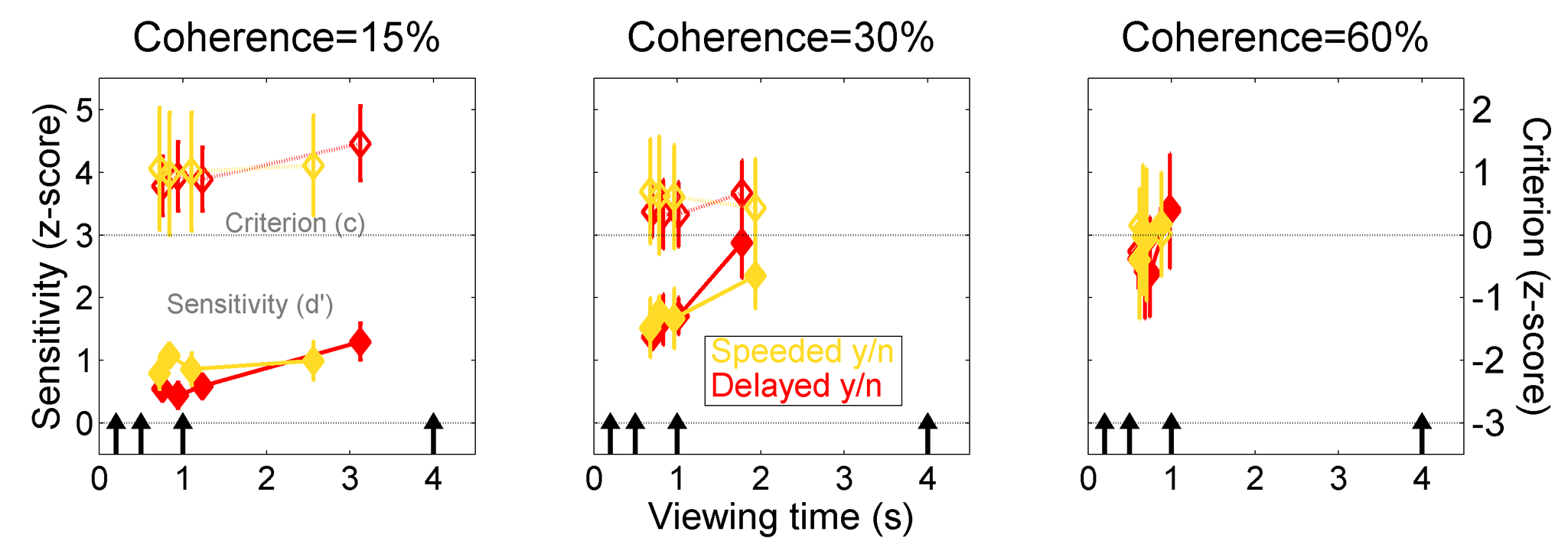

Supplement: Figure S4 — Control experiment. Sensitivity (d', continuous lines) and criterion (c, dotted lines) in the yes/no task. The red symbols (delayed y/n) refer to a condition in which the y/n response was given after the motion discrimination task, a condition that mimicked the main experiment. The yellow symbols (speeded y/n) refer to a condition in which the same observers were forced to give the y/n response immediately after the imperative cue (in place of the motion discrimination response). The mean discrimination rate in the delayed y/n condition was 77%, 89%, 96%, respectively for 15%, 30%, and 60% coherence. Same conventions as in Figure 3D–F of the main text. (TIF) [file pone.0017079.s004.tif]
